# Hermetic Welding of an Optical Fiber Fabry–Pérot Cavity for a Diaphragm-Based Pressure Sensor Using CO_2_ Laser

**DOI:** 10.3390/s22134700

**Published:** 2022-06-22

**Authors:** Hui Zhang, Yi Li, Zhuo Zhang, Chaoming Yang, Mingshan Liang, Yong Hu, Heming Wei, Fufei Pang

**Affiliations:** 1 Xi’an Modern Control Technology Research Institute, Xi’an 710065, China; xaxdliyi@163.com (Y.L.); lbczz0823@163.com (Z.Z.); 15529290898@163.com (C.Y.); 17795832146@163.com (M.L.); 2 Key Laboratory of Specialty Fiber Optics and Optical Access Networks, Shanghai University, Shanghai 200444, China; yonghu123@shu.edu.cn (Y.H.); hmwei@shu.edu.cn (H.W.); ffpang@shu.edu.cn (F.P.)

**Keywords:** optical fiber sensor, Fabry–Pérot cavity, pressure sensing, CO_2_ laser welding

## Abstract

A diaphragm-based hermetic optical fiber Fabry–Pérot (FP) cavity is proposed and demonstrated for pressure sensing. The FP cavity is hermetically sealed using one-step CO_2_ laser welding with a cavity length from 30 to 100 μm. A thin diaphragm is formed by polishing the hermetic FP cavity for pressure sensing. The fabricated FP cavity has a fringe contrast larger than 15 dB. The experimental results show that the fabricated device has a linear response to the change in pressure, with a sensitivity of −2.02 nm/MPa in the range of 0 to 4 MPa. The results demonstrate that the proposed fabrication technique can be used for fabricating optical fiber microcavities for sensing applications.

## 1. Introduction

Compared with traditional pressure sensors, optical fiber Fabry–Pérot interferometers (FPIs) have the advantages of high sensitivity, high precision, compact size, anti-electromagnetic interference, etc., and are widely used for the safety detection of nuclear reactors, aerospace, and underground gas wells [1,2,3,4,5], etc. Many optical fiber FP-based pressure sensors have been proposed based on various materials and fabricating technologies. Among them, the diaphragm-based extrinsic Fabry–Pérot interferometer (EFPI) is the most typical pressure sensor as it can be used for remote and highly sensitive sensing. It contains a thin film that is sensitive to change in the external pressure as the pressure can cause deformation on the film and then cause changes in the cavity length or phase of the light wave.

In order to increase the deformation sensitivity for pressure sensing, different materials have been proposed for the fabrication of diaphragms, including polymer [6,7,8], metal [9], silica [10,11,12,13,14], monocrystalline silicon [15], and sapphire [16,17], etc., which are usually fabricated by arc welding [10,11], CO_2_ laser welding [12,13], femtosecond laser precision processing [14], and bonding [16,17], etc. Among these sensors, the polymer-based diaphragm presents a high sensitivity to gas pressure, which is attributed to the small Young’s modulus [6]. However, the polymer diaphragm generally also demonstrates high sensitivity to temperature, which results in the temperature cross-sensitive issue. In addition, the polymer diaphragm is not suitable for high-temperature conditions. Metal films, as sensitive diaphragms, usually require vapor deposition methods to realize the coating process [9], and then can be formed as a cavity bonded with UV-curable adhesives, which makes the manufacturing process more complex. Relatively, all silica structures potentially enhance the sensor stability and can work under high-temperature conditions. However, the fabrication challenge of the thickness of the silica diaphragm being met is vital to the sensitivity.

Many methods have been proposed for silica-based FP cavities. One of them, the arc welding method, needs multiple welding steps to make a hermetic sensing cavity [10], of which the diameter and thickness may be limited. Traditional CO_2_ laser welding methods usually need two or more welding steps in order to fabricate such hermetic diaphragm-based sensors. In addition, their welding processes require precise and complex control operation [12,13]. The femtosecond laser machining method also requires etching of the sensing cavity of the FP sensor [14], and then welding of the sensor diaphragm to realize the hermetic cavity. The bonding process requires a high temperature of more than 1000 °C and a pressure of more than 10 MPa for several hours [16,17]. In order to ensure the bonding strength of the bonded sensor sample, it is also necessary to ensure a vacuum environment is maintained during the bonding experiment, which increases the manufacturing process complexity and difficulty of the sensor.

In this paper, a pressure sensor based on an improved CO_2_ laser welding method is proposed. This method can form a hermetic fiber FP cavity using only one-step welding. The welding parameters are easy to control through the laser power and welding time. It not only greatly simplifies the manufacturing process of the hermetic FP cavity but also avoids the limitation that the diameter of the sensitive film is smaller than the optical fiber and the non-uniformity of the sensing cavity caused by chemical corrosion. In addition, the fused silica film is selected in this work due to its temperature-tolerant feature. Since the sensor head is made from fused silica fibers and capillary, the problem with the coefficient of thermal expansion (CTE) mismatches is eliminated. J. Xu et al. demonstrated a similar sensor head with entire fused silica, which allowed high-temperature operation up to at least 700 °C [12]. Thus, the fabricated diaphragm-based fiber pressure sensor in this work has potential applications in high-temperature environments.

## 2. Principle and Simulation Analysis

### 2.1. Interference Principle of the FP Cavity

**Figure 1:**
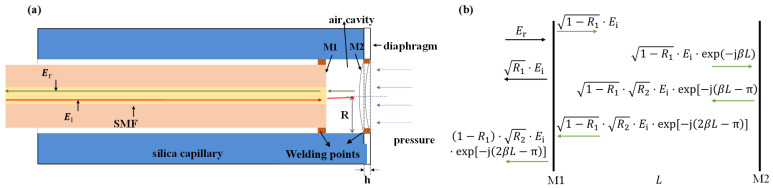
Diaphragm-based pressure sensor: (**a**) The structure of the sensor; (**b**) interference principle of the FP cavity.

The structure of the sensor in this study is shown in Figure 1a, which is composed of a single-mode fiber (SMF), fused silica capillary, and fused silica film. The SMF is used for signal light transmission. The capillary is used to integrate the SMF and silica film, which is used for sensing external pressure change.

When the beam of signal light with an electric field intensity of $E_{i}$ is incident from the optical fiber, reflection and refraction will occur at the interface M1 between the optical fiber and the air cavity and the interface M2 between the air cavity and the diaphragm, respectively. The transmission process is shown in Figure 1b. For convenience, the transmission loss in the cavity caused by the diffraction of the transmission medium and the transmission loss caused by the surface defect on the reflecting surface are not considered. At room temperature, the refractive index of the SMF ($n_{f}$) at 1550 nm is 1.45, and the refractive index of air ($n_{a}$) is 1. Then, the reflection coefficients at the interface M1 and M2 are $R_{1}=R_{2}={\left(n_{f}-n_{a}\right)}^{2}/{\left(n_{f}+n_{a}\right)}^{2}=0.034$. Considering that the reflectivity of the M1 and M2 interface is relatively low, the multiple reflected light can be ignored. Finally, the multi-beam interference model of the FP cavity is simplified to a two-beam interference model, and the total electric field intensity of reflected light is:

(1) $$E_{r}=\sqrt{R_{1}}\cdot E_{i}+(1-R_{1})\cdot \sqrt{R_{2}}\cdot E_{i}\exp[-j(2\beta L-\pi )]$$

where β is the optical fiber propagation constant, and $\beta =2\pi n_{a}/\lambda$. The total reflection spectrum can be calculated using Equation (2):

(2) $$R_{r}(\lambda )={\left|\frac{E_{r}}{E_{i}}\right|}^{2}=R_{1}-2\cdot (1-R_{1})\cdot \sqrt{R_{1}\cdot R_{2}}\cdot \cos(\frac{4n_{a}\pi L}{\lambda })+{\left(1-R_{1}\right)}^{2}\cdot R_{2}$$

It can be seen from Equation (2) that the reflection spectrum $R_{r}\left(\lambda \right)$ is only related to the cavity length *L* when *n_f_* and *n_a_* are fixed. If the cavity length changes, the wavelength of the reflection spectrum will move accordingly. Therefore, the external pressure can be measured by monitoring the shift in the resonant wavelength.

### 2.2. Sensing Principle of the FP Cavity

As shown in Figure 1a, when external pressure is applied, the silica film of the sensor is deformed, resulting in a change in the FP cavity length. The relationship between the change in the cavity length at the center point ΔL and the external pressure ΔP meets [18,19]:

(3) $$\Delta L=\frac{3{\left(1-\mu \right)}^{2}R^{4}}{16Eh^{3}}\cdot \Delta P$$

where E and μ are the Young’s modulus and Poisson coefficient corresponding to the silica film material, respectively. R is the effective radius and h is the thickness of the silica film.

### 2.3. Simulation of the FP Cavity Sensor

**Figure 2:**
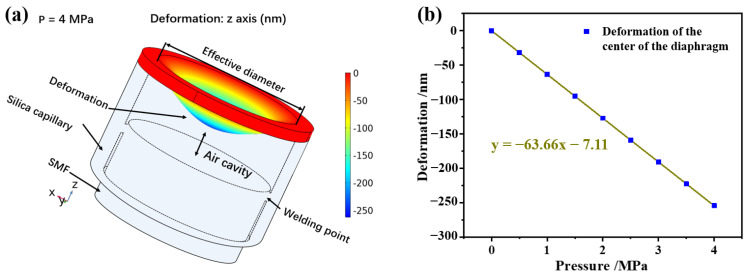
The physical model of the pressure senor. (**a**) The status of the pressure sensor under 4 MPa; (**b**) the deformation of the diaphragm center with pressure.

In this study, finite element analysis (COMSOL multiphysics) is used to investigate the pressure characteristics of the pressure sensor at room temperature. Firstly, the physical model of the sensor is built, and the cavity length of the sensor is set at 50 μm. The thickness and the radius of the silica diaphragm are 10 and 65 μm, respectively. Secondly, the pressure is changed from 0 to 4 MPa, and the pressure increment is set at 0.5 MPa in the simulation. Figure 2a shows the state of the sensor under a pressure of 4 MPa, and the maximum deformation at the center of the diaphragm is 254.7 nm. The deformation scale factor of the silica diaphragm is set at 100 in the COMSOL software to ensure the deformation is clear. Figure 2b shows the change curve of the shape variable at the center of the diaphragm with pressure. Through linear fitting, it is found that the sensitivity in the cavity length change of the sensor is −63.66 nm/MPa.

**Figure 3:**
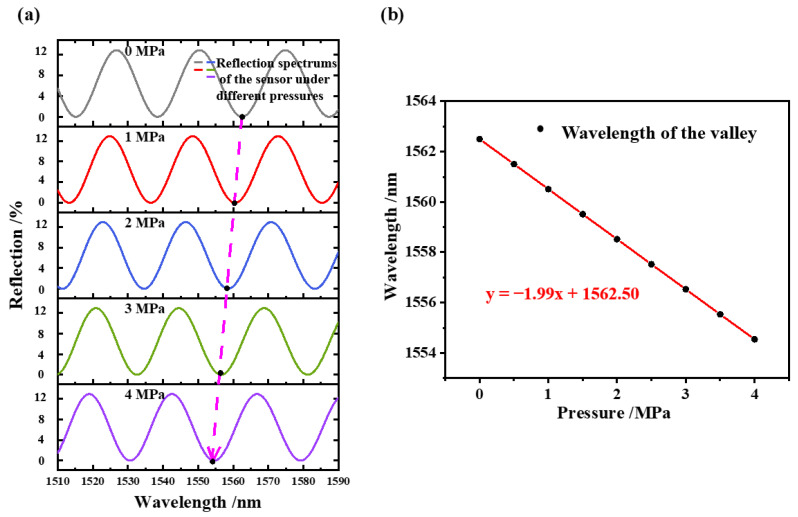
The simulated reflective spectrum of the sensor under different pressures. (**a**) The blue-shifted tendency when increasing the pressure from 0 to 4 MPa; (**b**) dip wavelength variation with increasing pressure.

According to the variation in the FP cavity length under different pressures, the shift trend of the wavelength of the reflection spectrum can be simulated using Equation (2). Figure 3a shows the simulation results of the reflection spectrum shift of the sensor under several different pressures from 0 to 4 MPa. It can be observed that the spectrum is blue-shifted as the pressure is increased. By tracking the resonant wavelengths near 1550 nm under different pressures, the variation curve of the wavelength with pressure can be obtained, as shown in Figure 3b. It can be seen that the wavelength versus the applied pressure is linear, and the sensitivity is calculated as −1.99 nm/MPa.

## 3. Analysis of Experimental Results

### 3.1. Fabrication of the Pressure Sensor

**Figure 4:**
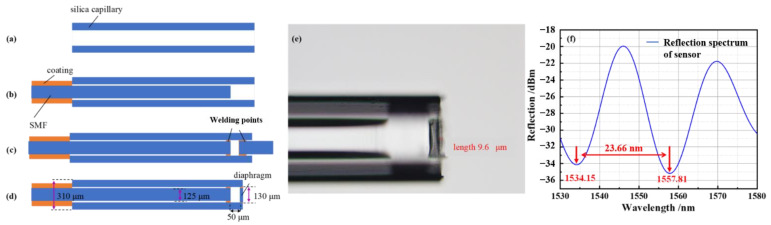
Fabrication of the diaphragm-based FP pressure sensor. (**a**–**d**) Steps of sensor fabrication; (**e**) the sensor’s graph under a microscope; (**f**) the reflection spectrum of the pressure sensor.

A CO_2_ laser welding machine (LZM100, Fujikura, Tokyo, Japan) and a polishing machine (MS08, westel, Shanghai, China) were used for the FP cavity sensor fabrication. The fabrication process of the sensor is mainly divided into the following steps: Firstly, a silica capillary with a length of about 5 cm, and inner and outer diameters of 130 and 310 μm were prepared, as shown in Figure 4a. A section of SMF with a certain length was then cleaned and cut to obtain a flat optical fiber end face, which was inserted into the capillary at the proper position (Figure 4b). Next, another SMF with a flat end face was placed on the right clamp of the welding machine for fixation, as shown in Figure 4c. The distance between the end faces of the two optical fibers, i.e., the FP cavity length, was set at 50 μm. Here, the FP cavity length was adjusted by the motors in the CO_2_ laser welding machine. The welding of the FP cavity was completed by the one-step light output of the CO_2_ laser. The fabrication of the sensor was preliminarily completed in this step. In order to improve the sensitivity of the sensor, the pressure sensing film of the sensor was polished by a polishing machine using diamond polishing papers with different grades of 30, 15, and 3 μm. Finally, the fabrication of the diaphragm-based sensor was finished, as shown in Figure 4d. Figure 4e shows a micrograph of the pressure sensor. The film thickness measured by a microscope (BX53M Olympus, Tokyo, Japan) was about 9.6 μm. The length of the air cavity was about 50.2 μm. Thinner films can be obtained with finer-grade polishing papers. Equation (4) is the calculation equation of the free spectral range *FSR*:

(4) $$FSR=\frac{\lambda^{2}}{2n_{a}L}$$

where λ is the working wavelength. It was calculated that FSR=23.92 nm, corresponding to a length of the FP cavity of 50.2 μm when the working wavelength was 1550 nm. Figure 4f shows the reflection spectrum of the pressure sensor. It was measured that FSR=23.66 nm from the spectrum, which is close to the theoretical calculation. The reflection spectrum presents relative low finesse, which is due to the optical loss factor and the imperfect reflectivity of the FP cavity. On the one hand, due to the diffraction effect, part of the reflected beam cannot be received by the SMF, which is equivalent to the transmission loss in the FP cavity [20]. On the other hand, there exists imperfect parallelism between the inner-side face and the SMF end face, as shown in Figure 4e. It introduces minor deviations in the propagation direction of the reflected beam, which is equivalent to the decrease in the reflectivity. As a result of the above reasons, the reflected spectrum of the FP cavity presents a deteriorated performance regarding the fringe contrast and fineness compared to an ideal FP cavity [20]. From the difference between the maximum and the minimum values of the reflection spectrum in Figure 4f, the contrast was calculated to be 15 dB, which provides enough clear fringe to characterize the pressure sensor.

In order to explore the influence of the length of the FP cavity on the effect of the welding on the FP cavity completed by CO_2_ laser welding, we carried out one-step welding experiments under different cavity lengths. The length of the FP cavity was changed from 30 to 110 μm with an interval of 10 μm.

**Figure 5:**
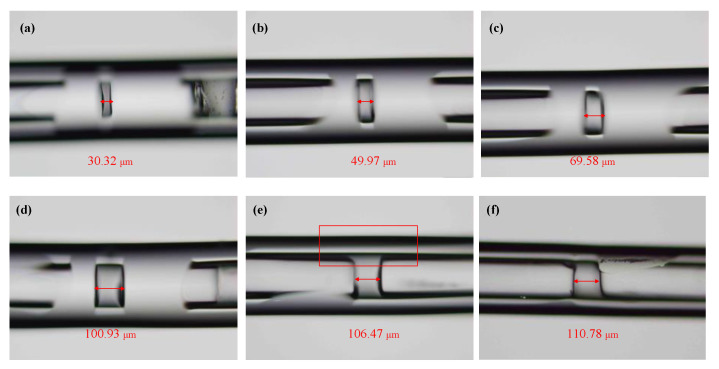
Micrographs of the sensing cavity welded by a CO_2_ laser for one time. (**a**) The cavity length was set at 30 μm; (**b**) 50 μm; (**c**) 70 μm; (**d**) 100 μm; (**e**) 110 μm with an increase in the welding power of the CO_2_ laser; (**f**) 110 μm with an increase in the welding time of the CO_2_ laser.

The experimental results show that the sealing of the cavity can be completed by one-step CO_2_ laser welding when the length of the FP cavity is less than 100 μm. Figure 5a–c shows the effect of the one-step welding of the FP cavity with lengths of 30, 50, and 70 μm, respectively. When the length of the FP cavity was set at approximately 100 μm, the sealing of the sensing cavity was still completed by one-step CO_2_ laser welding under a laser power of $P_{1}$ and welding time of $t_{1}$, as shown in Figure 5d. The silica capillary at the FP cavity bulged slightly, and the sealing of the sensing cavity could not be realized when the length of the sensing cavity was set at approximately 110 μm under the same welding parameters as when the length of the sensing cavity was 100 μm, as shown in Figure 5e. Under the same laser power of *P*_1_, the sensing cavity still could not be hermetically sealed if the welding time was increased further.

The experimental results show that the maximum cavity length of the sealed sensing cavity completed by one-step CO_2_ laser welding is 100 μm when the inner and outer diameter of the fused silica capillary is 130 and 310 μm, respectively. The sensing cavity still could not be sealed when the welding time and welding power of the CO_2_ laser was increased and the cavity length was larger than 100 μm. This is due to the temperature of the action area of the CO_2_ laser decreasing gradually away from the center, so that the silica capillary cannot be transformed into a molten state to complete the sealing welding with the SMF. Although the welding time of the CO_2_ laser increases, the welding quality of the sealed sensing cavity becomes significantly poorer.

### 3.2. Experiment and Analysis of Gas Pressure Sensing

**Figure 6:**
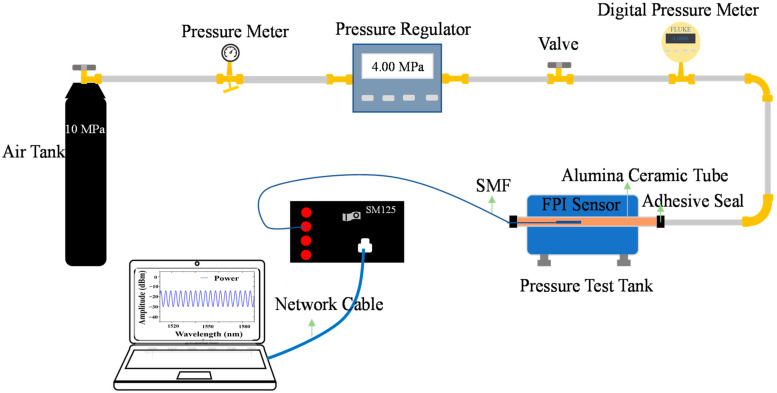
Schematic diagram of the experimental system for the pressure test experiment.

Figure 6 shows the pressure test system. A nitrogen cylinder provided gas pressure for the system. A pressure regulator (QY-C1-10-Y, resolution ± 0.02 MPa, Festo (China), Shanghai, China) was used to control the pressure in the environment where the sensor was located. A digital pressure gauge (700G08, fluke, Everett, WA, USA) was used to accurately display the pressure of the current sensor environment, with a resolution of 0.0001 MPa. The whole system was connected with 304 stainless steel pipes for the gas pressure conduction, and the sensor was placed in the steel pipe. An optical fiber sensing analyzer (SM125, Micron Optics Inc., Atlanta, GA, USA) was used for the reflection spectrum analysis, with a wavelength range from 1510 to 1590 nm and a resolution of 0.005 nm. A computer was used to display and process the reflection spectrum of the sensor.

**Figure 7:**
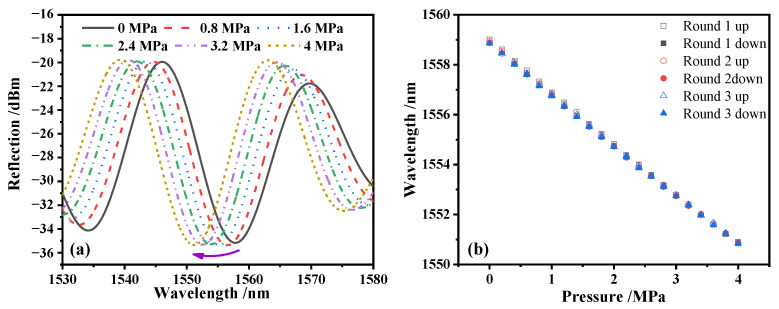
The reflection spectrum of the sensor under different pressures. (**a**) The spectrum shifting trend during rising pressure; (**b**) the three rounds’ sensitivity curve during the rising and dropping pressure.

The responses of the sensor with rising and dropping pressure were tested for three rounds within a range of 0 to 4 MPa with a step of 0.2 MPa. Figure 7a shows the blue-shift trend of the reflection spectrum as the pressure increased from 0 to 4 MPa, which was due to the compressing of the FP cavity by the increasing external pressure. The response of the resonant wavelength at 1557.81 nm for the three rounds are plotted in Figure 7b. Within the range of 0 to 4 MPa, the sensor manifests good linearity and repeatability.

In order to avoid the error caused by the sensor response determined by the single round experiment, the working line $y_{LS}=a+b\times p$ of the sensor was comprehensively evaluated using the results of the three rounds of pressure rising and falling experiments. Firstly, the calibration curve of the sensor needed to be calculated. $\bar{y_{i}}$ is the mean value of six identical detection points in the $i_{th}$ three rounds of pressure rising and falling experiments, and the curve determined by $\bar{y_{i}}$ is the calibration curve of the pressure sensor.

After obtaining the calibration curve of the pressure sensor, the slope b and intercept a of the working line $y_{LS}$ were calculated using the least square method, as shown in Equations (5) and (6) [21], and the slope b is the pressure sensitivity of the sensor:

(5) $$a=\frac{\sum_{i=1}^{m}p_{i}^{2}\cdot \sum_{i=1}^{m}{\bar{y}}_{i}-\sum_{i=1}^{m}p_{i}\cdot \sum_{i=1}^{m}{\bar{y}}_{i}\cdot p_{i}}{m\cdot \sum_{i=1}^{m}p_{i}^{2}-{\left(\sum_{i=1}^{m}p_{i}\right)}^{2}}$$

(6) $$b=\frac{m\sum_{i=1}^{m}{\bar{y}}_{i}\cdot p_{i}-\sum_{i=1}^{m}p_{i}\cdot \sum_{i=1}^{m}{\bar{y}}_{i}}{m\cdot \sum_{i=1}^{m}p_{i}^{2}-{\left(\sum_{i=1}^{m}p_{i}\right)}^{2}}$$

where m is the number of total detection points. There were 11 detection points in the range of 0 to 4 MPa with an interval of 0.4 MPa. Finally, it was determined that the working line of the sensor is $y_{LS}=1558.84-2.02\times p$, and the sensor sensitivity is −2.02 nm/MPa, which is close to the simulation result of the pressure sensor with a film thickness of 10 μm.

## 4. Conclusions

In this study, the CO_2_ laser welding technology was used for fabrication of the hermetic FP cavity through one-step welding, which simplified the preparation process of the hermetic FP cavity, and the film thickness was about 10 μm using the process of physical polishing. By exploring the influence of the length of the FP cavity on the effect of the welding on the FP cavity completed by CO_2_ laser one-step welding, it was concluded that the maximum length of the hermetic FP cavity was 100 μm through CO_2_ laser one-step welding. The working line of the sensor was determined through three rounds of rising and dropping pressure experiments, and the pressure sensitivity of the sensor was −2.02 nm/MPa, which was consistent with the simulation results. Thanks to the all-fused-silica structure of the sensor, the sensor can work stably under high temperatures and is expected to be used in harsh environments, such as nuclear reactors, internal combustion engine combustion chambers, etc.

## Data Availability

Not applicable.
